# Clinical and Radiological Outcomes of Anchored Stand-Alone Cage Compared to Conventional Plating in Multilevel Anterior Cervical Discectomy and Fusion: A Systematic Review

**DOI:** 10.7759/cureus.72386

**Published:** 2024-10-25

**Authors:** Bishoy Ghobrial, Alexander Price, Jacques Pretorius, Hussam Elkhwad

**Affiliations:** 1 Trauma and Orthopaedics, University Hospital Galway, Galway, IRL

**Keywords:** anchored, anterior, anterior plate construct, arthrodesis, cervical discectomy, fusion, plate, stand-alone

## Abstract

Multilevel cervical degenerative disc disease (cDDD) is typically treated through anterior cervical discectomy and fusion (ACDF). Traditionally, the plate-cage construct (PCC) has been utilized, though alternatives such as the locking stand-alone cage (LSC) have become popular. This systematic review aims to assess differences in clinical and radiological outcomes between LSC and PCC methods in the ACDF management of multilevel cDDD by aggregating existing literature. A comprehensive search of five electronic databases (PubMed, Medline, Ovid, Embase, and Cochrane) was conducted from 2015 to 2022 following the Preferred Reporting Items for Systematic Reviews and Meta-Analyses (PRISMA) guidelines. The comparative studies were assessed using the Methodological Index for Non-randomized Studies (MINORS) criteria and the two randomized controlled trials (RCTs) were assessed using the Jadad scale for RCT. Eight studies met the inclusion criteria, comprising a total cohort of 516 contiguous multilevel cDDD cases treated via ACDF, with 254 (49%) managed with PCC and 262 (51%) treated with LSC. The comparative outcomes assessed included the rate of subsidence and the rate of postoperative dysphagia. The LSC approach exhibited a higher incidence of cage subsidence compared to PCC. Conversely, dysphagia was observed more frequently in patients receiving PCC compared to those treated with LSC. Clinical outcomes and functional scores did not reveal significant differences. Clinical measures such as the Japanese Orthopaedic Association (JOA) score, Neck Disability Index (NDI), visual analog scale (VAS), and Odom's and Robinson's criteria revealed no significant differences between treatment groups in seven of the studies. However, one of the included studies reported a statistically significant improvement in the LSC group for VAS and Odom's criteria. The incidence of cage subsidence in ACDF surgery varies depending on the cage type employed. Among the reviewed studies, the LSC group exhibited a higher incidence of subsidence compared to the PCC group, with the exception of one study that reported no cases of subsidence. Variability in subsidence rates across studies may be attributed to differences in surgical techniques, patient demographics, and follow-up periods. Further research is necessary to investigate associated risk factors and to develop strategies aimed at minimizing this complication in ACDF procedures.

## Introduction and background

Cervical spondylosis is a frequent condition that can present with either radiculopathy or myelopathy [1]. General management typically begins with conservative therapies, although anterior cervical discectomy and fusion (ACDF) is an effective option for refractory cases [2]. The outcomes of ACDF depend on factors such as the extent of decompression, stability of the fused segment, restoration of cervical lordosis, and the avoidance of complications. The plate-cage construct (PCC) technique is widely accepted for its significant immediate stabilization of the surgical site, preventing graft dislocation and subsidence prior to fusion [3]. However, it is associated with various complications, with postoperative dysphagia, hematoma, and recurrent laryngeal nerve palsy being the most common [4]. The utilization of a PCC appears to also be associated with significant adverse events, including dysphagia, neurovascular injuries, and adjacent segment degeneration, among others [5].

When a plate construct is not utilized, stabilization of the interbody cage can be achieved through locking screw systems associated with locking stand-alone cages (LSCs) [6]. The LSC method was developed to minimize these complications while retaining the advantages of interbody cage insertion during ACDF. LSC may be favorable due to its smaller surgical field and lower dissection volume when compared to PCC. It is observed that ACDF is inherently associated with complication risks, which may increase with multiple levels involved; thus, while the LSC may provide non-inferior clinical outcomes compared to PCC in single-level cervical degenerative disc disease (cDDD) managed by ACDF, the same may not hold true for multiple-level cases [7,8]. The aim of this systematic review is to compare clinical and radiological outcomes between LSC and PCC techniques in ACDF for the management of multilevel cDDD, with a specific focus on evaluating the incidence of cage subsidence and the rate of postoperative dysphagia.

## Review

Methods

Search Strategy

Our search strategy was based on the PICO (population, intervention, comparison, and outcomes) format to assess whether patients with multilevel cDDD treated by ACDF (population) with LSC (intervention) differ from those treated with PCC (comparator) in terms of clinical and radiological outcomes (outcome), following the Preferred Reporting Items for Systematic Reviews and Meta-Analyses (PRISMA) guidelines and recommendations [9]. Electronic searches were conducted across PubMed, Medline, Ovid, Embase, and Cochrane databases. Searches were conducted across all databases covering literature published from January 2015 to December 2022. This date range was chosen to focus on the most recent evidence and outcomes. MeSH strategies were utilized with keywords including anterior, cervical discectomy, fusion, arthrodesis, stand-alone, anchored, zero-profile, and plate. Two investigators were involved in reviewing the retrieved articles for further identification of relevant studies.

The search strings used are as follows: ("anterior cervical discectomy and fusion" OR "ACDF") AND ("multilevel" OR "multiple levels" OR "two-level" OR "three-level") AND ("stand-alone cage" OR "zero-profile plate" OR "anchored cage") AND ("plate-cage construct" OR "conventional ACDF" OR "anterior plate" OR "PCC") AND ("clinical outcomes" OR "radiological outcomes" OR "subsidence" OR "dysphagia") ("cervical degenerative disc disease" OR "cDDD") AND ("anterior cervical discectomy and fusion" OR "ACDF") AND ("multilevel") AND ("stand-alone" OR "zero-profile" OR "anchored") AND ("outcomes" OR "results" OR "complications").

Filters were used to restrict studies to those that included human studies, published in English and within the specified date range.

Selection Criteria

Inclusion and exclusion criteria were established to ensure relevant studies were selected. Inclusion criteria encompassed studies published in English, involving adult participants aged 18 to 80 years, involved humans, and were indexed in PubMed, Medline, Ovid, Embase, and Cochrane databases, and consisted of randomized controlled trials (RCTs) that directly compared LSC and PCC in multilevel ACDF during the specified period. Exclusions eliminated studies (i) not conforming to these characteristics, (ii) including non-English publications, (iii) abstract-only studies, (iv) lower evidence levels, specifically level 5 studies, (v) studies not indexed in PubMed, (vi) articles that included potential conflicts of interest, and (vii) RCTs comparing LSC with PCC in a single level, segmental, and non-contiguous ACDF. This aimed to ensure the inclusion of high-quality studies that directly addressed the research question.

Data Extraction

From the included studies, data were independently extracted by two reviewers, focusing on the Japanese Orthopaedic Association (JOA) scores, Neck Disability Index (NDI), visual analog scale (VAS), dysphagia occurrence, and cage subsidence rates. Basic study details, including first author, publication year, design, country, surgical level, sample size, gender distribution, mean age, and follow-up duration were also recorded. Absolute values were tabulated and compared between the two reviewers. No differences were identified between the findings of the two reviewers.

Quality Assessment

Two reviewers independently assessed the risk of bias in the selected studies. The six comparative studies were assessed using the Methodological Index for Non-randomized Studies (MINORS) [10], as shown in Table 1. The two RCTs were assessed using the Jadad scale for RCTs [11,12].

**Table 1:** Methodological Index for Non-randomized Studies (MINORS).

Study	Shi et al. (2015) [13]	Y Chen et al. (2016) [14]	Perrini et al. (2017) [15]	Yun et al. (2017) [6]	Zhu et al. (2019) [16]	Z Chen et al. (2021) [17]
Clearly stated aim	2	2	2	2	2	2
Inclusion of consecutive patients	2	2	2	2	2	2
Prospective collection of data	0	0	0	0	0	0
Endpoints appropriate to the aim of the study	1	1	2	2	2	2
Unbiased assessment of the study endpoint	1	1	1	1	1	1
Follow-up period appropriate to the aim of the study	2	2	2	2	2	2
Loss to follow up less than 5%	2	2	2	2	2	2
Prospective calculation of the study size	0	0	0	0	0	0
An adequate control group	2	2	2	2	2	2
Contemporary groups	2	2	2	2	2	2
Baseline equivalence	1	1	1	1	1	1
Adequate statistical analyses	2	1	2	2	2	2
Sum	17	16	18	18	18	18

Results

Search Results

**Figure 1:**
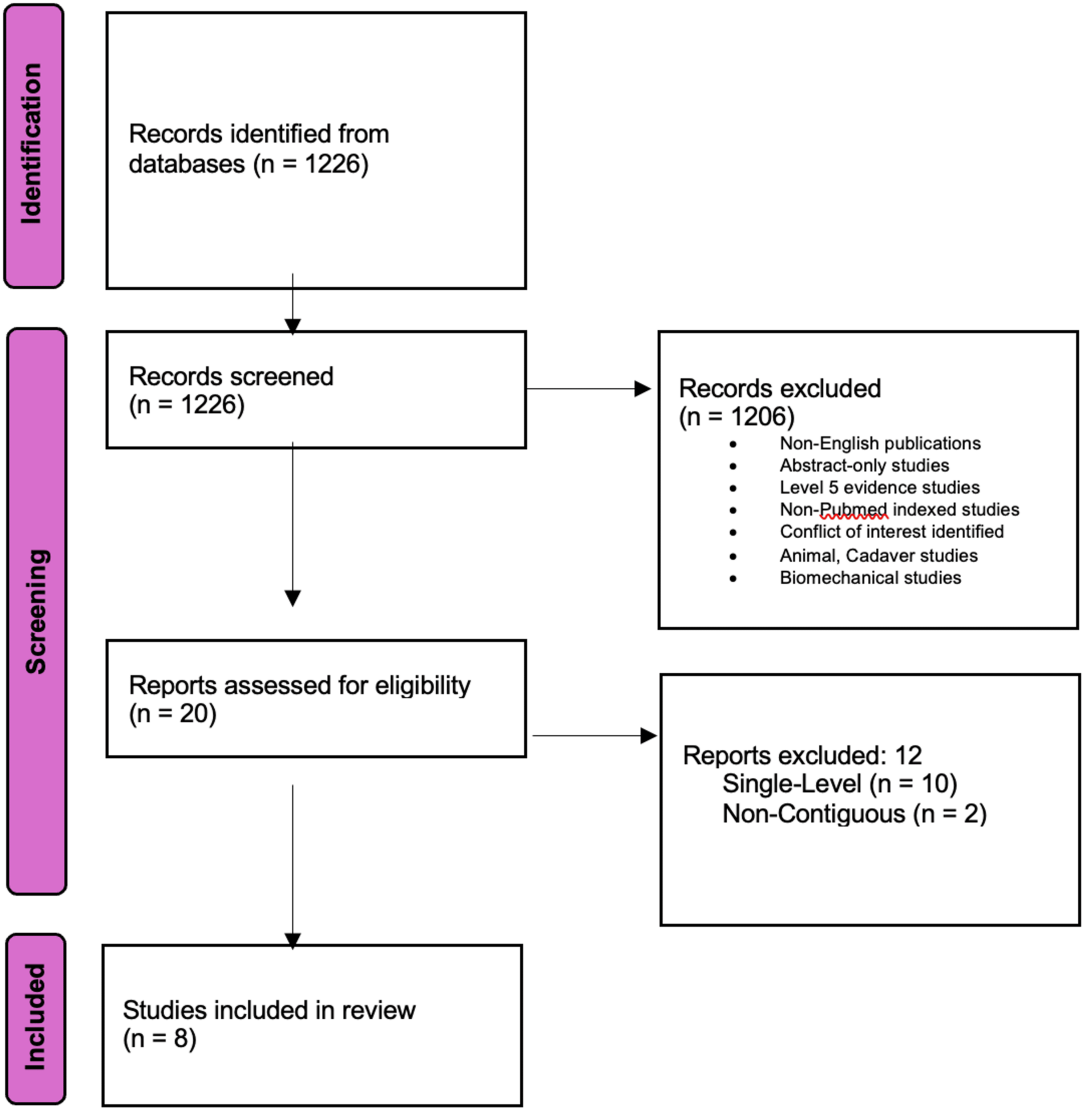
Flow diagram of search strategy results conducted per Preferred Reporting Items for Systematic Reviews and Meta-Analyses (PRISMA) guidelines. PRISMA [18].

A thorough literature search yielded 1226 records. No additional records were identified from alternate sources. Following the screening process, 1206 records were excluded based on predetermined criteria. After assessing full-text articles for eligibility, 12 additional studies were excluded for focusing on single-level or non-contiguous ACDF, 10 studies and two studies, respectively. This did not align with the research objective. Consequently, eight studies were included in this systematic review. This would provide valuable insight into the comparison of LSC and PCC in multilevel ACDF. The selection process ensured a robust analysis of the available evidence. The flow diagram of the search strategy can be visualized below in Figure 1.

Demographics

Demographically, the selected studies encompassed a total of 516 patients, with 255 undergoing the PCC procedure, including 156 males and 99 females, and 261 receiving the LSC intervention, including 149 males and 112 females. Gender distribution was comparable, indicating no significant differences in demographics, with an average age of 58 years for both groups (Table 2). A statistical comparison of demographic variables across the studies showed no significant differences between the groups in terms of age, gender distribution, and follow-up duration. The mean age between the two groups was comparable, with the PCC group being 56.2 ± 3.9 years versus the LSC group being 55.6 ± 4.8 years, with no statistically significant difference (p = 0.189). The follow-up duration did not differ significantly between the groups either, with the PCC group being 30.1 ± 5.1 months and the LSC group being 30.5 ± 4.9 months (p = 0.868). With regards to gender distribution, the ratio between males and females was similar between the two groups, with a chi-square test revealing no significant difference (χ² = 0.73, p = 0.393). The results indicate that the demographic variables were well-matched across the two study groups, minimizing potential confounding effects due to differences in baseline characteristics. Overall, these findings indicate that the demographic characteristics between the LSC and PCC groups were comparable with no significantly significant difference observed.

**Table 2:** Study demographics. PCC: plate-cage construct; LSC: locking stand-alone cage; R: retrospective; P: prospective; OS: observational study; CA: comparative analysis; CS: case series; RCT: randomized controlled trial; NR: not reported.

Study	Design	Overall	PCC					LSC				
		Size (n)	Size (n)	Age	Male	Female	Follow-up	Size (n)	Age	Male	Female	Follow-up
Shi et al. (2015) [13]	R, OS	38	20	56.7±3.9	12	8	30.1±2.8	18	56.2±4.8	11	7	30.5±3.4
Y Chen et al. (2016) [14]	R, CA	72	38	56.2±5.7	25	13	36	34	56.9±5.9	21	13	36
Perrini et al. (2017) [15]	R, OS	78	22	55.6±7.8	11	11	33.6±12.8	56	51.1±10.4	31	25	30.55±13.1
Yun et al. (2017) [6]	R, OS	63	32	54.2±9.9	29	3	NR	31	53.3±7.6	22	9	NR
He et al. (2018) [12]	P, RCT	104	52	59.5±12.6	27	25	24	52	55.4±12.4	28	24	24
Zhu et al. (2019) [16]	R, OS	62	32	55.3±13.1	18	14	36	30	56.6±12.4	16	14	36
Scholz et al. (2020) [11]	P, RCT	41	20	58	13	8	24	21	58	11	9	24
Z Chen et al. (2021) [17]	R, CS	58	38	56.9±10.7	21	17	31.1±16.9	20	53.9±16.9	9	11	30.5±16.1
		516	255	58	156	99		261	58	149	112	

Clinical Outcomes

Several studies investigated the clinical outcomes of various interventions for spinal disorders. Y Chen et al. found no significant differences in subjective patient-reported outcomes among groups [14]. Similarly, Shi et al. and He et al. found no statistically significant difference in JOA and NDI scores [12,13]. However, Yun et al. reported statistically significant differences favoring the cage-alone group in VAS (p = 0.022) and Odom's criteria (p = 0.032) [6]. Perrini et al. found no statistically significant difference in Robinson criteria and NDI outcomes [15]. Scholz et al. also reported no significant difference in NDI and Odom’s criteria [11]. Zhu et al. showed no difference in JOA and NDI scores and no difference in VAS at three months postoperatively, but the difference was more pronounced in the cage-alone group at the three-year follow-up (p > 0.05) [16]. Finally, Z Chen et al. found no statistically significant difference in NDI and JOA outcomes [17].

Postoperative Dysphagia

**Figure 2:**
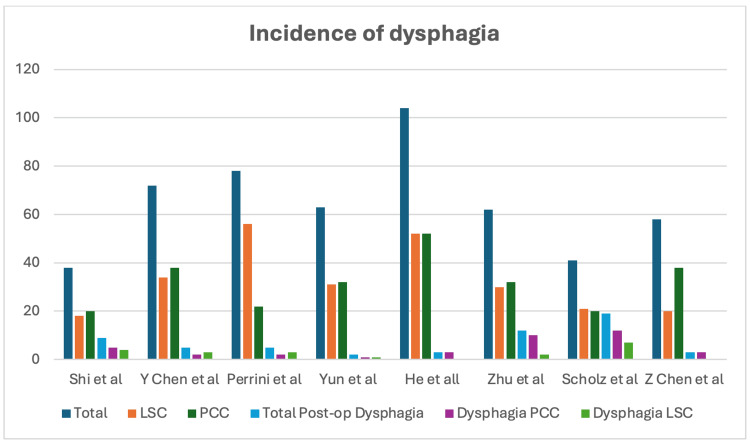
Incidence of postoperative dysphagia. LSC: locking stand-alone cage; PCC: plate-cage construct. References: Shi et al. (2015) [13]; Y Chen et al. (2016) [14]; Perrini et al. (2017) [15]; Yun et al. (2017) [6]; He et al. (2018) [12]; Zhu et al. (2019) [16]; Scholz et al. (2020) [11]; Z Chen et al. (2021) [17].

Based on the eight articles, it appears that the overall incidence of postoperative dysphagia after multilevel ACDF with PCC versus LSC is relatively low. However, there is some variation in the reported incidence rates between the studies. Overall, it appears that the incidence of postoperative dysphagia is relatively low after both PCC and LSC, although the PCC group may have a slightly higher incidence in some studies. It is important to consider that other factors, such as surgical technique and patient selection, may also influence this complication. This is illustrated in Figure 2.

Cage Subsidence

**Figure 3:**
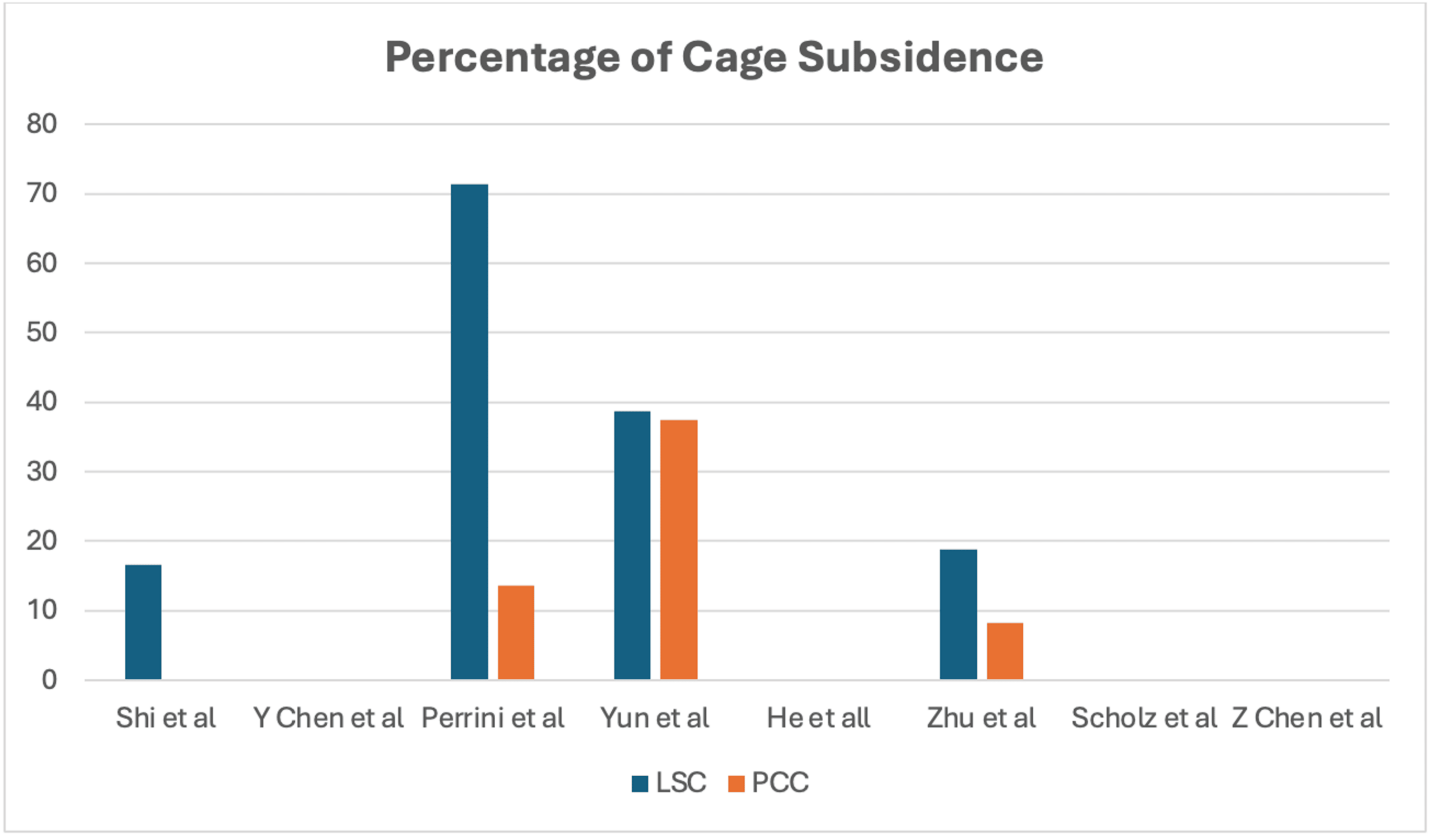
Percentage of cage subsidence. LSC: locking stand-alone cage; PCC: plate-cage construct. References: Shi et al. (2015) [13]; Y Chen et al. (2016) [14]; Perrini et al. (2017) [15]; Yun et al. (2017) [6]; He et al. (2018) [12]; Zhu et al. (2019) [16]; Scholz et al. (2020) [11]; Z Chen et al. (2021) [17].

Cage subsidence is another complication of concern. Three of the included studies did not record the occurrence of cage subsidence. Shi et al. showed no incidence in the PCC group, but had 16.7% in the LSC group [13]. Perrini et al. reported a subsidence rate of 13.67% in the PCC group and 71.42% in the LSC group [15]. Yun et al. recorded a subsidence rate of 37.5% in the PCC group and 38.7% in the LSC group [6]. Zhu et al. reported a subsidence rate of 18.8% for the LSC group and 8.3% for the PCC group [16]. Z Chen et al. reported no cases of subsidence in both groups [17]. This is illustrated in Figure 3.

Discussion

Multilevel cervical spondylotic myelopathy is a complex degenerative condition that predominantly affects patients older than 45 years, often associated with advanced age, hyperostosis, and/or repetitive injuries or abnormal motions [19]. Patients typically present with myelopathy, along with neck and shoulder pain. Surgical intervention has proven effective in alleviating symptoms and maintaining long-term improvements [20]. ACDF has been established as a "criterion standard" surgical technique since its introduction by Muheremu et al. in 1955 [21]. The evolution of instrumentation and implants has enhanced the ability to better correct cervical alignment, create broader interbody fusion beds, and improve postoperative biomechanics through the use of a PCC or integrated fixation implants [22].

However, studies reveal that the application of a multilevel PCC necessitates larger surgical exposure and increased soft tissue retraction. This can elevate the risk of injury to surrounding structures, including the esophagus, trachea, vasculature, and neural elements [23]. Damage to the esophagus or recurrent laryngeal nerve may lead to complications such as dysphagia and dysphonia [24]. Various studies have established a connection between PCC usage and these complications. While ACDF can be conducted at both single and multiple levels, an increase in the number of fused segments correlates with a higher rate of operations [25].

There was variability noted among the studies included, where some indicated no significant differences in clinical outcomes, while others revealed advantages in select measures. Yun et al.’s findings suggest potential superiority for the LSC approach in specific outcomes [6]. The heterogeneity among these results emphasizes the need for a more thorough investigation and consideration of factors influencing the selection of interventions for spinal disorders. Assessment of clinical outcomes should encompass both subjective patient-reported outcomes and specific measures, like VAS, JOA, and NDI. Furthermore, long-term follow-up may expose differences in outcomes that were not initially evident.

Dysphagia is one of the most frequently reported complications following ACDF, with variability in reported incidence likely due to the heterogeneity of current literature [26]. While some prospective studies have documented rates as high as 71%, this review found that the overall incidence of postoperative dysphagia was relatively low for both PCC and LSC constructs, although some studies indicated a slightly higher incidence in the PCC group [27]. It is essential to account for additional factors, such as surgical technique and patient selection, that may influence dysphagia rates.

The overall low incidence of postoperative dysphagia suggests that both PCC and LSC constructs are generally safe options for multilevel ACDF. Nonetheless, careful assessment and individualized decision-making are crucial, considering specific patient characteristics and surgical expertise. Further research, particularly larger-scale studies, is warranted to yield more comprehensive data on potential factors contributing to postoperative dysphagia in ACDF using diverse cage constructs.

Cage subsidence is a noteworthy phenomenon that can occur in ACDF, particularly with LSC constructs. Concerns regarding cage subsidence have led surgeons to lean toward PCC over alternative options like LSC. A systematic review indicated that cage subsidence occurs in approximately 21% of patients [28]. The risk of subsidence appears lower with the use of polyether ether ketone (PEEK) or titanium cages when additional screws are utilized. The existing literature does not sufficiently evaluate whether subsidence correlates with clinical outcomes.

This review provided valuable insights into the incidence of cage subsidence and reinforced the notion that PCC may be a more favorable choice compared to LSC for multilevel ACDF procedures. The 0% subsidence rate reported by Shi et al. in the PCC cohort suggests superior stability and a reduced risk of subsidence [13]. In contrast, higher subsidence rates in the LSC group raise concerns regarding its suitability for multilevel applications [6,15]. The preference for PCC can be attributed to its enhanced stability and lower subsidence risk due to the supportive plate component.

Future research should aim to evaluate subsidence risk further, including its clinical significance and the relationship between subsidence and both material characteristics and dimensional aspects of cages. A more comprehensive understanding of these variables will help inform clinical decision-making in cervical spine surgeries.

This systematic review provides valuable insights into the management of multilevel cDDD but does have a few limitations. These limitations include a limited number of studies being included in the review, with only eight studies meeting the inclusion criteria and therefore this may be insufficient to generalize these findings to all patients undergoing an ACDF. The heterogeneity among studies may have varied in terms of patient demographics, surgical techniques used, follow-up periods, and outcome measures used. The variability in surgical techniques and surgeon experience may have impacted the outcomes and rates of complications. Although the review aims to minimize any bias, the potential for included studies to be subject to bias still exists. This may be in the form of selection bias, reporting bias, or publication bias.

## Conclusions

This systematic review shows that both the LSC and PCC methods provide similar outcomes, as evidenced by comparable scores in patient-reported outcome measures such as JOA, NDI, and VAS scores. The incidence of postoperative dysphagia was relatively low in both groups, although a marginally higher incidence was noted in the PCC group. Surgical technique and patient selection were key influencing factors regarding postoperative dysphagia. A significant finding of this review was the higher incidence of cage subsidence in the LSC group compared to the PCC group. This finding suggests that the PCC method may potentially offer greater stability and a lower risk of subsidence in multilevel ACDF, potentially making it a more favorable option. The review acknowledges the limitations, including the limited number of studies and the heterogeneity among the included studies, which may influence the generalizability of the findings to the general population.

Further studies, in particular larger scale studies, are recommended to assess the long-term outcomes and to better understand the factors contributing to complications such as postoperative dysphagia and radiological cage subsidence. Further studies should aim to assess the clinical significance of cage subsidence and the material characteristics of the cages being used, the surgical techniques and operative times, and the surgeon's experience, which may affect clinical and radiological outcomes. In summary, while both the LSC and PCC are effective methods in ACDF, PCC may offer advantages in reducing cage subsidence.
